# Breeding Cultivars for Resistance to the African Sweetpotato Weevils, *Cylas puncticollis* and *Cylas brunneus,* in Uganda: A Review of the Current Progress

**DOI:** 10.3390/insects14110837

**Published:** 2023-10-25

**Authors:** Benard Yada, Paul Musana, Doreen M. Chelangat, Florence Osaru, Milton O. Anyanga, Arnold Katungisa, Bonny M. Oloka, Reuben T. Ssali, Immaculate Mugisa

**Affiliations:** 1 National Crops Resources Research Institute (NaCRRI), NARO, Kampala 999123, Uganda; 2 Department of Horticultural Science, North Carolina State University, Raleigh, NC 27695, USA; 3 International Potato Center, Kampala 999123, Uganda; 4 Department of Agricultural Production, Makerere University, Kampala 999123, Uganda

**Keywords:** sweetpotato, weevils, *Cylas* spp., resistance, breeding, Uganda

## Abstract

**Simple Summary:**

Sweetpotato, a very important food and income security crop in Uganda, is severely damaged by African species of sweetpotato weevils, often causing unacceptable yield losses, particularly in susceptible varieties and areas that are prone to drought. The management of sweetpotato weevils is difficult due to their concealed feeding behavior. Various methods have been applied to manage sweetpotato weevils, including chemical methods, cultural practices, biological control, and host plant resistance, but with limited success so far. Host plant resistance could be an effective approach to managing sweetpotato weevils. As such, the National Crops Resources Research Institute has developed and released sweetpotato varieties that show varying levels of resistance for the farming community in Uganda and the sub-Saharan African region. Here, we review the progress that has been made so far towards developing sweetpotato weevil resistance and related research on weevil management in Uganda.

**Abstract:**

In sub-Saharan Africa, sweetpotato weevils are the major pests of cultivated sweetpotato, causing estimated losses of between 60% and 100%, primarily during dry spells. The predominantly cryptic feeding behavior of *Cylas* spp. within their roots makes their control difficult, thus, host plant resistance is one of the most promising lines of protection against these pests. However, limited progress has been made in cultivar breeding for weevil resistance, partly due to the complex hexaploid genome of sweetpotato, which complicates conventional breeding, in addition to the limited number of genotypes with significant levels of resistance for use as sources of resistance. Pollen sterility, cross incompatibility, and poor seed set and germination in sweetpotato are also common challenges in improving weevil resistance. The accurate phenotyping of sweetpotato weevil resistance to enhance the efficiency of selection has been equally difficult. Genomics-assisted breeding, though in its infancy stages in sweetpotato, has a potential application in overcoming some of these barriers. However, it will require the development of more genomic infrastructure, particularly single-nucleotide polymorphism markers (SNPs) and robust next-generation sequencing platforms, together with relevant statistical procedures for analyses. With the recent advances in genomics, we anticipate that genomic breeding for sweetpotato weevil resistance will be expedited in the coming years. This review sheds light on Uganda’s efforts, to date, to breed against the *Cylas puncticollis* (Boheman) and *Cylas brunneus* (Fabricius) species of African sweetpotato weevil.

## 1. Introduction

Sweetpotato (*Ipomoea batatas* (L.) Lam.) (2n = 6x = 90) is the third most important root crop globally after potato and cassava, with an estimated total global production of 88.7 million metric tons [1]. It is a major staple food in sub-Saharan Africa (SSA), where it ranks as the second most important root crop after cassava. Africa is currently the second-largest sweetpotato-producing continent after Asia, with an estimated annual production of 28.6 million metric tons, accounting for 32% of its entire production worldwide [1]. This clonally propagated crop is part of the Convolvulaceae family, and it is the only species of the Ipomoea genus that is of economic importance [2].

Sweetpotato weevils (SPWs), *Cylas* spp., are the main pests that affect sweetpotatoes in Uganda and the SSA region, causing estimated yield losses that range from 60% to 100% [3,4,5], mainly during dry spells and under heavy infestations. *C. puncticollis* and *C. brunneus* are the predominant species occurring in Africa [3,6]. Adult *Cylas* spp. attack the leaves, stems, and storage roots of sweetpotatoes, whereas the larvae feed and grow within the storage roots and stems. This leads to poor-quality roots with an unacceptable smell and bitter taste due to the secretion of defense sesquiterpenes by the infested plants, which makes the roots unfit for human and livestock consumption [7,8]. It is reported in *C. formicarius* that the secretion of defense sesquiterpenes is due the formation of an ethylene dark brown necrotic layer within the sweetpotato storage root tissue as result of the injury caused by adult and larvae feeding [9].

The concealed feeding behavior of weevils within storage roots makes them quite challenging to control using methods such as chemical control. Host plant resistance, therefore, presents a more suitable alternative for the integrated pest management (IPM) of this pest. Sweetpotato breeding, however, remains a difficult task due to the large and complex nature of its genome, with additional limitations such as self and cross-incompatibility, limited flowering ability, and seed setting [10], which further complicate conventional breeding. 

Attempts at breeding SPW-resistant cultivars have not yielded much to date, due to the lack of significant heritable resistance in the sweetpotato germplasms that have been examined previously [11,12]. However, some landraces with moderate resistance have previously been identified, and researchers are currently using these as parental materials in an effort to develop resistant varieties [13,14]. Sweetpotato was termed as an “orphan crop” with limited private investment in the development of its genomics infrastructure [15]. Genomic selection and marker-assisted selection stand out as promising options for developing SPW-resistant varieties in Uganda [14]. This review gives an insight into the progress that has been made so far in cultivar resistance breeding against the African sweetpotato weevils, *C. puncticollis* and *C. brunneus*, in Uganda. It also highlights aspects relating to the management and control of the two *Cylas* species. 

## 2. Taxonomy and Geographical Distribution of *Cylas* Species

Sweetpotato weevils (*Cylas* spp.) belong to the Order Coleoptera, Brentidae family, and Cyladinae subfamily [16]. They are divided into three species; *C. brunneus*, *C. puncticollis*, and *C. formicarius*, which vary in physical characteristics such as body form, hind femoral length, and genitalia features, among others [3]. *C. formicarius* is typically an Asian species, but it also occurs in other tropical and sub-tropical parts of the world like Europe, North America, and Africa [17]. *C. puncticollis* and *C. brunneus*, however, are restricted to Africa [3,18]. They are the most prevalent and commonly reported SPW species on the continent [13,19,20,21]. *C. puncticollis*, in particular, is the most abundant in tropical Africa, reported in 24 African countries, including Uganda, Rwanda, Ghana, Nigeria, DR Congo, Kenya, Malawi, Cameroon, and Ethiopia [8,22].

## 3. Description and Life Cycle of *Cylas* Species

**Figure 1:**
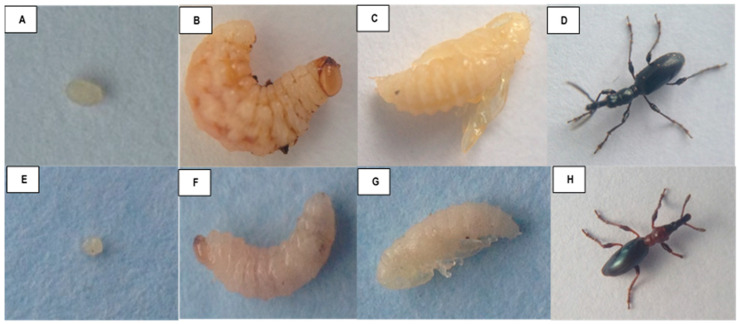
Growth stages of the two African sweetpotato weevil species, *C. puncticollis* ((**A**)—egg; (**B**)—larvae; (**C**)—pupa; and (**D**)—adult) and *C. brunneus* ((**E**)—egg; (**F**)—larvae; (**G**)—pupa; and (**H**)—adult).

Adult females of the *Cylas* species usually lay their oval, yellowish eggs singly in sweetpotato roots or stems. They then seal the cavity with a fecal plug [23]. *Cylas* spp. usually prefer to lay their eggs within the roots, mostly near the roots’ crown. The egg stage lasts between 5 and 12 days, with *C. puncticollis* eggs maturing much quicker than those of *C. brunneus* [24]. The larvae of *Cylas* spp. are usually curved in shape and white in color, feeding and developing within the stems and roots of sweetpotato. The pupae are glabrous in shape, with those of *C. puncticollis* being relatively larger than those of *C. brunneus* [23] (Figure 1). Male and female adults of both *Cylas* species can be distinguished using the shapes of their distal antennal segments, notably filiform in males and club-like in females [16]. The two *Cylas* species differ in their morphology, coloration, and general development (Table 1). 

## 4. Host Range and Dispersal of *Cylas* Species

*Cylas* spp. have a number of wild host plants, mainly other Ipomoea species. These include: morning glory (*I. eriocarpa*), beach morning glory (*I. pes-caprae* (L.)), water spinach (*I. aquatic*), wild potato vine (*I. pandurate*), and cotton (*Gossypium hirsutum* (L.)) [7,8]. However, the ideal host plant for *C. puncticollis* and *C. brunneus* is sweetpotato. In Uganda, the demand for additional hosts for weevil survival may not be as great, because most cropping systems allow for year-round sweetpotato production. Additionally, the weevil population growth rate on other Ipomoea species that are wild hosts may be very low, because they do not produce swollen roots that serve as oviposition sites. Both adult *C*. *puncticollis* and *C*. *brunneus* are usually dispersed by flying and crawling, with males being more agile and flying more than females. Most females are dispersed more through crawling than flying [7,23]. Long-distance dispersal, however, occurs mostly through the movement of infested sweetpotato storage roots and vines [8].

## 5. Damage Caused by *Cylas* Species

**Figure 2:**
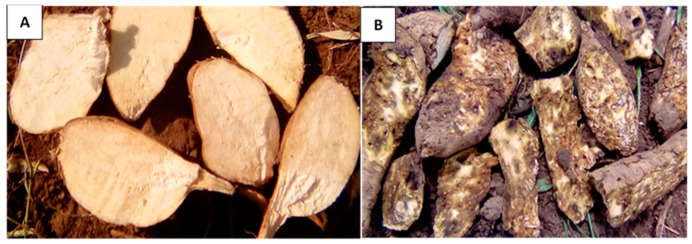
Sweetpotato storage roots shortly after harvest: (**A**)—clean (no weevil damage) and (**B**)—weevil-infested (visible damage).

Adults injure the vines and leaves by feeding on the epidermis, scraping and creating oval patches, which may lead to a significant reduction in plant vigor and poor establishment in the case of early infestation [3]. Larvae usually damage the stem by feeding at the base, thereby affecting the vascular system, causing the vines to swell and eventually wilt or die [8]. However, the most significant damage is inflicted when weevils attack the underground storage roots. The feeding punctures that adults make on the exteriors of root surfaces are distinguishable from oviposition sites by their higher depth and the lack of a fecal plug [25]. Larval feeding and tunneling creates irregular galleries in the inner part of the root or stem, further damaging the plant (Figure 2). In response to this, the roots produce sesquiterpenes in self-defense, which give the roots an unacceptable odor and bitter taste, rendering them unsuitable for consumption either as food or animal feed [8,12]. The damage caused by *Cylas* spp. therefore affects the quantity, quality, and value of the sweetpotato storage roots produced. Under severe infestations in SSA, losses in commercial storage root yield of up to 60–100% have been documented [3,26].

## 6. Management and Control Strategies

Several measures have been proposed for the management, control, and eradication of SPWs. (Table 2). Because none of them are completely effective in the control of this elusive pest, an integrated pest management (IPM) approach is usually recommended. Control is made difficult because all immature developmental stages of *Cylas* spp. occur deep inside the storage roots and stems of the plant [8].

## 7. Mechanisms of Resistance of Sweetpotato to *Cylas* spp.

Different resistance mechanisms found existing in sweetpotato germplasms against SPWs have previously been reported, including antibiosis, antixenosis, and escape [46]. Other studies have demonstrated that SPW resistance is more than simply an escape mechanism through field phenology such as deep-rooting plants, but rather an active mechanism that is quantifiable and potentially manageable for breeding purposes [11,12,21]. Hydroxycinnamic acid (HCA) esters in the root latex and root surface (epidermal and peridermal tissues) of sweetpotatoes have been shown to deter the development of SPW larvae, reducing feeding damage and oviposition. The HCAs were identified as heptadecylcaffeic acid, hexadecylcaffeic acid, octadecylcaffeic, hexadecylcoumaric acid, octadecylcoumaric acid, and 5-0-caffeoylquinic acids from the Ugandan sweetpotato varieties “New Kawogo” and “Tanzania” [12,13,14]. These acids are a major group of phenolic compounds with bioactive properties that are produced by plants for protection against biotic and abiotic stress [47].

## 8. Progress in Breeding for Resistance to *Cylas* spp.

Progress in breeding for sweetpotato weevil resistance has been rather slow, owing to the complexity of the sweetpotato genome and the limited sources of genetic resistance to *Cylas* spp. [11,12]. Genetic studies in sweetpotato are limited by high levels of self and cross-incompatibility, limited flowering ability, high ploidy levels, and poor seed germination, as also observed in Taiwan [48]. Each successful cross usually leads to the production of less than three botanical seeds [49]. This presents a challenge for conventional breeding that solely relies on hybridization to produce genetic variability.

Nevertheless, the breeding program at the National Crops Resources Research Institute (NaCRRI) in Uganda has in the past, and to date, used conventional breeding and population improvement through recurrent mass selection for variety development in Uganda [50,51]. The phenotyping of SPW resistance was based on severity and incidence estimations. Sweetpotato severity was routinely assessed using standard protocols and a scale of 1–9 was employed where 1 = no damage; 3 = minor; 5 = moderate; 7 = heavy; and 9 = severe damage, with numbers in between representing intermediate ratings [52]. The incidence of sweetpotato weevil damage was measured as a percentage of the number of storage roots showing weevil damage symptoms to the total number of roots in a plot.

In the past, sweetpotato morphological traits such as vine vigor, ground cover, foliage weight, storage root stalk (neck length), storage root cortex thickness, storage root size, storage root shape, storage root formation (arrangement), root latex production, and dry matter content were documented to have a significant impact on enhancing SPW field resistance [43]. However, through a correlation and path coefficient analysis, neck length was identified as the key morphological SPW resistance predictor [53]. Clones with a long storage root neck length ensure the placement of storage roots deeper in the soil. This, in turn, helps to minimize the exposure of storage roots to SPW, thus reducing damage. This trait will be useful in selecting for SPW resistance among breeding clones during the early stages of breeding. This could be accomplished at a single location prior to advancing clones for multi-location tests and analyses of biochemical hydroxycinnamic acids, thereby minimizing research costs.

To date, the National Crops Resources Research Institute of the National Agricultural Research Organization (NARO) has released 29 sweetpotato varieties with varying levels of weevil resistance in Uganda [51,54,55]. All the 29 sweetpotato varieties that have been released in Uganda so far are either moderately resistant or susceptible to sweetpotato weevils (Table 3). Varieties like “New Kawogo”, which express a moderate resistance to *Cylas* spp., are currently deployed as parental material in SPW breeding and related studies in Uganda [12].

## 9. The Genetic and Biochemical Basis for Sweetpotato Weevil Resistance

The development of sweetpotato varieties with resistance to SPWs requires an understanding of the underlying biochemical and genetic mechanisms of resistance to this pest. Our understanding of the genetic and biochemical basis of resistance to SPWs has been improved in the past decade through a number of studies. Previous research conducted to determine the basis of weevil resistance in the Ugandan landrace, “New Kawogo”, to the African SPW *C*. *puncticollis* has revealed that resistance in this variety was not only active, but also quantifiable and manageable for breeding [12]. In another study, 134 sweetpotato cultivars and landraces were evaluated for weevil resistance. Some sweetpotato cultivars, including “New Kawogo”, expressed resistance to *Cylas* spp., while others such as “Tanzania” and “NASPOT1” turned out to be susceptible [21].

In order to identify the biochemical basis of resistance to SPWs, the performances of seven resistant varieties of sweetpotato were compared with three susceptible varieties in field trials and laboratory bioassays [13]. Using liquid chromatography-mass spectrometry (LC-MS) of root surface and epidermal extracts, significant variations in the concentrations of hexadecyl, heptadecyl, octadecyl, and quinic acid esters of caffeic and coumaric acid were exhibited, with higher concentrations of these compounds being correlated with SPW resistance. “New Kawogo” recorded the highest concentration of these compounds. It was, therefore, concluded that the selection of sweetpotato varieties with higher levels of HCAs, particularly in the storage root surface, would probably contribute to the development of resistance to SPWs. However, this would necessitate the screening of hundreds of breeding lines using freeze-dried samples of roots and analyses using LC-MS in order to achieve long-term population improvement. Developing such a chemotyping platform, though desirable, would be quite costly [14].

In a further effort to understand the segregation of HCA esters that confer resistance to SPWs, Anyanga et al. [11] quantified these chemicals among 287 progenies of a segregating bi-parental population of the “New Kawogo” × “Beauregard” (NKB) cross. Beauregard is a USA-released cultivar, highly susceptible to SPWs. The results revealed that HCA esters conferred resistance to SPWs and segregate in the F_1_, suggesting that they are controlled quantitatively, and thus might be useful as markers for resistance in sweetpotato breeding programs. The progeny that displayed more resistance to SPWs than “New Kawogo”, together with those that had higher concentrations of HCA esters, were selected as new candidates and are currently being utilized as parental genotypes in sweetpotato breeding.

Knowledge of the inheritance and heritability of resistance to African SPWs is also apparently still limited. Some studies have been conducted in the past to establish the heritability of SPW resistance, in which resistance to *Cylas* spp. was measured on the basis of the severity of weevil damage to field storage roots and total HCA ester concentrations [14]. A moderately high broad-sense heritability was estimated (*H*^2^ = 0.49). Another study revealed similar results (with *H*^2^ = 0.49) and further showed that additive effects contributed the most to SPW resistance and HCA concentration [56]. More recently, a narrow-sense heritability (*h*^2^ = 0.30) was reported for SPW resistance based on a relatively high population [45]. The adoption of modern experimental designs, such as row-column designs which use more replications for field phenotyping, will probably enable the estimation of higher heritability values for SPW resistance for future genomic analyses.

From these studies, the authors observed that 18 progeny exhibited positive transgressive segregation (performing better than the resistant parent “New Kawogo”, mean severity 2.1) for field-based SPW resistance, with mean severity scores below 2.1. For HCA-based resistance, two progeny outperformed the resistant parent “New Kawogo” (HCA concentration 282 ng/g) for total HCA concentration. This implied that SPW resistance was heritable and could be improved in populations through crossing.

## 10. Marker-Assisted Selection and Genomic Selection for SPW Resistance

The limited number of genomic resources has been a major bottleneck in breeding for SPW resistance. Over the years, sweetpotato genomic improvement has generally lagged behind other important crops such as maize [57], rice [58], and wheat [58]. This has been attributed to the scarcity of molecular markers to facilitate genetic analyses [59], the lack of appropriate specific statistical procedures due to its polyploid nature, and limited user-friendly computer software for implementing the statistical procedures for genetic analyses of important traits [15]. To maximize the success rate in developing sweetpotato varieties, genomic tools are critical [60], particularly since most traits in sweetpotato are quantitatively inherited [59,61].

Molecular markers have hardly been used in sweetpotato genetic research compared to other staple crops, yet they are critical in the genetic analysis of complex traits [59,62] and increase the efficiency of selection for low-heritability traits through marker-assisted selection (MAS) [59,62]. 

Sweetpotato weevil resistance, like most sweetpotato traits of agronomic and economic importance, is quantitative in nature, controlled by many genes and with a high environmental plasticity [49,57]. This has resulted in slow breeding progress through the use of conventional breeding approaches. Associating such a complex trait with genomic regions can be performed through QTL analyses [63]. However, there is currently a relatively low number of published simple sequence repeats (SSR) and single-nucleotide polymorphism (SNPs) markers for sweetpotato [14,57,64,65]. In the past, most molecular genetic studies in sweetpotato have been based on dominant markers that were easier to score [66,67,68], but with limited utility for sweetpotato improvement via MAS and genomic selection (GS), unlike SSRs and SNPs [14,69].

Some initial progress has been made in identifying SSR and SNP markers for use in breeding for SPW resistance. Yada et al. [70] conducted a study in which he examined the genetic diversity in the NKB population using SSR markers. The same population was later genotyped with these SSR markers to identify SPW resistance loci [14]. Five SSR markers were observed to be associated with field SPW severity, whereas seven were linked to HCA ester concentration. Three markers showed a significant association, suggesting their high potential for use in the development of SPW-resistant cultivars [14]. Four QTLs were later identified using next-generation sequencing (NGS) technology, explaining a total of 37.7% of the variation in SPW resistance and 15.9% of the variation in storage root HCA ester concentration in the NKB population [57].

Genomic selection stands out as a novel approach to accelerating genetic gains in sweetpotato [71]. In their pioneering work to explore the potential of genomic selection in sweetpotato, the predictive ability based on several factors was performed for simple traits and yield traits for a single population. Though the predictive ability was low for complex traits, the use of multi-trait models offered confidence for the better prediction of such traits [71]. In addition, studies on other polyploids like octaploid strawberry have reported a high accuracy for lower-heritability traits [72,73]. Despite the reports of lower heritability for insect pest resistance across crop species, the application of genomic prediction in maize showed a moderate to high predictive ability for fall army worm and maize weevil [74]. Therefore, the application of genomic selection for weevil resistance is feasible and can be achieved after optimizing the size, composition, structure, and phenotyping of the training population and assembling sufficient diversity in the population.

## 11. Genomic Resources for Sweetpotato Improvement

Through global collaborative research, the high-quality sequencing of the genomes of two diploid close relatives of sweetpotato, *I. trifida* and *I. triloba*, was successfully accomplished [75] and a genome sequence-based marker platform for sweetpotato improvement was developed. From these genome reference sequences, thousands of single-nucleotide polymorphism (SNP) markers have been developed and are being used for selecting key traits such as beta carotene content in breeding populations in Africa. *Ipomoea trifida* is the closest wild species to sweetpotato, as evidenced by a number of cytology and molecular studies [76,77,78,79]. Though it is the preferred reference for *I. batatas*, the *I. triloba* genome can also be used as a complementary reference sequence in sweetpotato breeding [75]. In 2017, the first sweetpotato reference genome was released by Yang et al. [80] using a haplotyping method. Nevertheless, there are still on-going efforts to develop a high-quality whole-genome sequence for hexaploid sweetpotato [75,80]. Once completed, this will facilitate genomic selection and enable the more efficient introgression of important traits such as SPW resistance through controlled crosses from genomics-based characterized parents carrying resistance alleles, ultimately leading to the development of sweetpotato varieties with desirable attributes.

The advances in DNA sequencing methods through NGS technology and bioinformatics resources over the past decade have also enabled large numbers of SNP markers to be developed and utilized in molecular breeding using different tools [75,80]. 

## 12. Genetic Transformation for Weevil Resistance

Research has been conducted in SSA to develop varieties that are resistant to SPWs through genetic transformation and crossing landraces with transgenic cultivars. One of the first transformations in sweetpotato was for weevil resistance. Agrobacterium-mediated transformation was used to introduce the delta endotoxin gene cry8Db, which was obtained from *Bacillus thuringiensis*, into the sweetpotato cultivar “KB1” [61]. This gene encodes a protein that is insecticidal and fatal to many insect larvae. A total of 21 lines were later regenerated from the calli that had been transformed. Upon testing, these lines later displayed a lower weevil infestation compared to those that were not transformed [81]. Agrobacterium-mediated transformation was also utilized to transform the Ugandan sweetpotato landrace “Kyebandula” by introducing the genes for the bacterial endotoxins cry7A1 and cry3Ca. Positive PCR findings purportedly showed that the procedure was successful. Nevertheless, the transformed calli were later unable to develop into whole plants [82].

Ekobu et al. [83] conducted an experiment in which they evaluated the toxicity of Cry proteins to *C. puncticollis* and *C. brunneus*. The three most active Bt Cry proteins with potential to confer resistance against *C. puncticollis* and *C. brunneus* were Cry7Aa1, cryET33-34, and Cry3Ca1 with an LC50 value (mg/mL diet) against the first instar *C. puncticollis* and second instar *C. brunneus* below 1 ppm. However, they were not effective against SPW larvae, a result partly attributed to the root tissue’s low level of Cry protein expression. Another study investigated the possibility of hybridizing transgenic and non-transgenic sweetpotatoes to produce transgenic offspring [40]. However, hybrid sweetpotato produced from the transgenic event did not exhibit any substantial variations in Cry protein expression. Transgenic sweetpotatoes that expressed Cry proteins did not exhibit the anticipated levels of *C. puncticollis* resistance, and a further evaluation of more transgenic events was recommended.

Recently, Anyanga et al. [84] studied the effect of combining the anti-feedant effects of HCA esters with the expression of Bt proteins in transformed plants. The study observed that, although resistance was naturally conferred by HCA acids and deterred oviposition by adults, these compounds were restricted to the roots and did not protect against the larvae, which feed in the cortex, causing the greatest damage. The authors evaluated the bioactivity of the Cry7Aa1 protein and HCA esters both individually and in combination for causing mortality against SPW larvae. Larval mortality proved to be higher when a combination of HCA esters and Bt protein was used under laboratory bioassay conditions. Further research was recommended to establish the effect of the combination under field conditions.

## 13. Future Prospects

Further research on genetic transformation and genome editing: The evaluation of more transgenic events carrying cry genes would increase the likelihood of successfully identifying those with a higher cry protein expression, which could potentially provide effective weevil control. Mitigating any existing constraints to genetic transformation could contribute to significant developments in the genetic enhancement of sweetpotato. The use of RNAi technology that has been tested on the African sweetpotato weevil species [85,86] offers future potential for managing this pest in Uganda. Nevertheless, it is time to apply modern approaches such as gene editing in sweetpotato for sweetpotato weevil management.

Improved phenotypic strategies and analytics: As sequencing and genotyping costs continue to drop, phenotyping has been highlighted as one of the most costly and major limitations to the precise mapping of traits in plant breeding [87]. Novel next-generation phenotyping platforms that facilitate the accurate, low-cost, and timely acquisition of phenotypic data [88] will need to be developed and utilized for enhancing the quality of the phenotypic data collected in Uganda and the region. High-throughput, near-infrared reflectance spectroscopy (NIRS) analyses of root chemistry composition, which have been implemented in many breeding programs [87], will need to be adopted and scaled out in the SSA region. Many imaging platforms and computer software have been developed for the automated screening of attributes such as root and leaf architecture, though most are currently not yet being utilized in sweetpotato research in the region [89,90,91]. Weevil severity in Uganda is presently still measured via visual assessments of damage symptoms on roots [92]. Additionally, the application of modern analytics is vital to maximizing phenotypic data outputs. For instance, the use of modern experimental designs, such as row-column designs, which cater for more replications for field phenotyping, and the analysis of data using mixed models would enhance the heritability values for genomic analyses.

Advances in plant bio-chemistry: Advances will need to be made in the use of plant biochemistry for revealing the basis of pest resistance in sweetpotato. Although plant biochemistry is now being used for screening for SPW resistance in Uganda breeding programs, low-cost analysis throughput platforms such as near-infrared spectroscopy (NIRS) are required, since wet chemistry platforms like liquid chromatography mass spectrometry (LC-MS) are still costly for chemical resistance profiling when several clones have to be screened at different breeding stages. The calibration of NIRS for the analysis of the total HCA ester concentrations in root samples would enhance the throughput screening of breeding materials for population improvement [14]. This could be plausible considering that a similar method, Fourier-infrared spectroscopy, has previously successfully been used for the quantification of the total HCA esters in forages [93].

Advances in genomics: It is currently more important than ever that more molecular markers, particularly SNPs, are identified through NGS platforms, since this would expedite genomic breeding for weevil resistance and other key traits in Uganda. This has been enabled by recent research advances such as the published genome sequence of diploid *I. trifida*, the closest relative and putative progenitor of sweetpotato [75]. Ultimately, the completion of the ongoing whole-genome sequencing of cultivated hexaploid sweetpotato in the near future will further enable the maximum exploitation of the genomics-assisted improvement of this crop in Uganda and the region.

Exploring the potential of heterosis based breeding: In the past, sweetpotato breeding has mainly relied on the ability of breeders to identify parental genotypes possessing desirable traits, and to combine these parents through hybridization schemes such as polycross and controlled cross nurseries [51,93,94,95]. Preliminary studies have, however, shown that there are better genetic gains from heterosis-based breeding compared to using polycross [94]. Nonetheless, the potential limitation of this approach is the high level of self and cross-incompatibilities in some of the diverse parental genotypes [96], in addition to seed set abortions.

Molecular methods are vital in sweetpotato improvement via heterosis. Genetic markers such as SNPs are important for diversity analyses of parental genotypes and for the selection of unrelated parents while constituting heterotic groups for use in population improvement [97]. Because of self-incompatibility and high levels of inbreeding depression in sweetpotato, heterotic groups are currently divided according to the long-term geographic adaptations of breeding parental genotypes such as the African, Asian, and South and North American heterotic groups [51]. The application of heterosis-based breeding, through selecting and genotyping the best-performing SPW-resistant clones and utilizing the superior unrelated parents for subsequent population improvement, would reduce the likelihood of recombining close parental genotypes, eventually leading to higher genetic gains.

## 14. Conclusions

Host plant resistance is a very promising approach for sweetpotato weevil management. However, attempts to develop resistant cultivars in Uganda have not yet yielded as much as anticipated. Some progress has nonetheless been made over the past decades, as evidenced by the moderately weevil-resistant varieties that are currently under production by farmers in Uganda and SSA. The underlying genetic and bio-chemical basis for resistance has been understood to some extent and molecular markers such as SNPs have been developed, though still not being fully utilized in breeding. More effort will be needed by sweetpotato breeders to develop additional markers for mapping sweetpotato weevil resistance to facilitate marker-assisted selection and genomic selection. With the ongoing sequencing of the hexaploid sweetpotato genome, we anticipate that genomics-assisted breeding for sweetpotato weevil resistance will be expedited in the coming years. Moreover, the global advancements in sweet potato phenomics and biochemistry will eventually bring forth cutting-edge approaches for sweetpotato improvement. This will eventually lead to accelerated genetic gains in breeding for resistance to sweetpotato weevils and other key traits in Uganda and the SSA region.

## Figures and Tables

**Table 1:** A comparison between *Cylas puncticollis* and *Cylas brunneus* [7,8,16].

Trait	*C. puncticollis*	*C. brunneus*
**Morphology**	Eggs: 0.45 × 0.30 mm^2^ (average size)	0.7 × 0.5 mm^2^ (average size)
	Larvae: 5–10 mm long	7–8 mm in length
	Pupae: 6–7 mm long, creamy-white cuticle	4–5 mm long, white in color
	Adults: 5–8 mm long, eyes narrowly parted	5–7 mm long, eyes widely separated
	Male: 6.9–6.7 mm long, filiform antennal	5.7–5.5 mm long, filiform antennal
	Female: 7.2–6.8 mm long, club-like antennal	5.7–5.5 mm long, club-like antennal
**Oviposition**	Females take 2–24 days before laying eggs	Females start laying eggs a day or so after becoming sexually active
**Development**	Takes place between 17.5–35 °C	Is possible between 17.5–32 °C
**Coloration of adults**	Initially creamy white, but later change to gray and black	Turn from creamy white to brown and finally black with reddish brown thorax
**Host range**	Has a wider host range including morning glory, cotton, sesame, and maize	Has a smaller host range including morning glory and water spinach
**Distribution**	Reported in 24 African countries	Recorded in 9 African countries
**Dispersion**	Adults can fly for longer distances (up to 120 m)	Adults fly for short distances (up to 80 m)

**Table 2:** An overview of the management and control strategies for *Cylas* species.

Cultural Practices
These practices contribute towards the reduction in weevil populations either by making sweetpotato plants less accessible to weevils or by preventing the weevil population from increasing to harmful levels [23]. They include using clean planting material, crop rotation [27], hilling up and mulching, the removal of alternate crops and wild host plants, field sanitation [25,28], the use of barrier crops, intercropping, the planting of new crop away from weevil-infested fields, timely or early planting and harvesting before onset of the dry season [22,29], and flooding [30]. Cultural interventions are most effective when weevil populations are low; they are less successful during extended droughts, which typically result in population peaks [23].
**Chemical Practices**
Insecticides as foliar sprays control weevils to some extent [31,32]. They can reduce the populations of adult weevils, but may not adequately control immature larvae due to their cryptic nature [3]. Dipping plant material into a synthetic pesticide prior to planting can delay pest invasion for a number of months [25]. Insecticides are expensive and at times inaccessible to growers in SSA, making their use impractical and unsustainable [33].
**Biological**
Natural enemies such as ants, maggots, and wasps attack weevils, but most seem to be ineffective at suppressing SPW populations under field conditions [23,34]. Entomopathogenic fungi such as *Beauveria bassiana* have successfully been used to control SPWs in combination with other control methods. They cause a reduction in feeding ability, fecundity, and egg viability in *C. puncticollis* [35]. Entomopathogenic nematodes like *Steinernema carpocapsae* and *Heterorhabditis bacteriophora* have shown potential for the practical biological suppression of *Cylas* spp., but are not readily available, and small-scale farmers may not have the required purchasing power [19,36]. Bacteria such as *Bacillus thuringiensis* Berliner (Bt) have been developed to confer inherent pest resistance against SPWs [37,38]. Bt sweetpotato events against SPWs have previously been tested in SSA, but with minimal success, though transgenic plants expressing Bt cry genes are in use in some countries outside the region [39,40]. A combination of the male annihilation technique (MAT) and the sterile insect technique (SIT) appears to be quite effective in the management of *Cylas* spp. They were successfully used in Japan to eradicate *C. formicarius* over a wide area [41,42].
**Host Plant Resistance**
This approach is a major line of defense against SPWs. It involves the use of resistant or tolerant clones. Various investigations have been carried out in the past to comprehend the mechanisms of SPW resistance, and efforts have been made to develop SPW-resistant varieties [11,12,13,43]. Sweetpotato landraces and varieties were found to be highly heterogeneous in their susceptibility to field infestations by weevils [21]. Deep-rooting and early-maturing varieties were less prone to infection than shallow-rooted and late-maturing types [44]. The low heritability of the weevil-resistant trait coupled with the limited number of varieties that exhibit high levels of SPW resistance have contributed to the slow progress in developing weevil-resistant cultivars in SSA through conventional breeding [12,45]. There are further ongoing efforts to develop clones with significant levels of resistance using genomic tools.

**Table 3:** Reaction of sweetpotato varieties released in Uganda to *Cylas* spp.

Year of Release	Variety	^a^ Reaction to SPWs
1995	“Bwanjule”	MR
	“New Kawogo”	MR
	“Sowola”	MR
	“Tanzania”	S
	“Wagabolige”	MR
	“Tororo 3”	MR
1999	NASPOT 1	S
	NASPOT 2	S
	NASPOT 3	MR
	NASPOT 4	MR
	NASPOT 5	MR
	NASPOT 6	MR
2004	“Ejumula”	S
	SPK004 (Kakamega)	S
2007	NASPOT 7	S
	NASPOT 8	S
	NASPOT 9 O	S
	NASPOT 10 O	S
	“Dimbuka Bukulula”	S
2010	NASPOT 11	S
2013	NASPOT 12 O	S
	NASPOT 13 O	S
2017	NAROSPOT 1	MR
	NAROSPOT 2	S
	NAROSPOT 3	S
	NAROSPOT 4	S
	NAROSPOT 5	S
2023	NAROSPOT 6	MR
	NAROSPOT 7 O	MR

^a^, level of resistance of sweetpotato varieties to sweetpotato weevil infestation in the field, S, Susceptible (mean sweetpotato weevil severity of 6–9); MR, moderately resistant (mean sweetpotato weevil severity of 3–5); and R, resistant (mean sweetpotato weevil severity of 1–2).

## Data Availability

The raw data sets of the reviewed papers in this article are available for public and can be accessed online at: https://sweetpotatobase.org/ (accessed on 4 October 2023).
